# Low-temperature studies of propene oligomerization in ZSM-5 by inelastic neutron scattering spectroscopy

**DOI:** 10.1039/c9ra03568k

**Published:** 2019-06-14

**Authors:** Alexander P. Hawkins, Andrea Zachariou, Paul Collier, Russell A. Ewings, Russell F. Howe, Stewart F. Parker, David Lennon

**Affiliations:** School of Chemistry, University of Glasgow Joseph Black Building Glasgow G12 8QQ UK David.Lennon@glasgow.ac.uk +44(0)-141-330-4372; UK Catalysis Hub, Research Complex at Harwell, STFC Rutherford Appleton Laboratory Chilton Oxon OX11 0FA UK; Johnson Matthey Technology Centre Blounts Court, Sonning Common, Reading RG4 9NH UK; ISIS Neutron and Muon Source, STFC Rutherford Appleton Laboratory Chilton Oxon OX11 0QX UK; Department of Chemistry, University of Aberdeen Aberdeen AB24 3UE UK

## Abstract

Observation of the oligomerization of propene in ZSM-5 at 293 K by neutron vibrational spectroscopy shows that the product species are linear alkyl chains. No evidence is found for the formation of branched products. The selective formation of linear alkyl chains is attributed to a confinement effect within the zeolite pore structure. A role for zeolite crystallite size, a controllable parameter within the catalyst preparative stage, in being able to influence the product composition in technically relevant olefin oligomerization reactions is considered.

## Abstract 2

The interaction between ZSM-5 and propene over the temperature range 140–373 K is investigated by application of inelastic neutron scattering spectroscopy.

## Graphical abstract

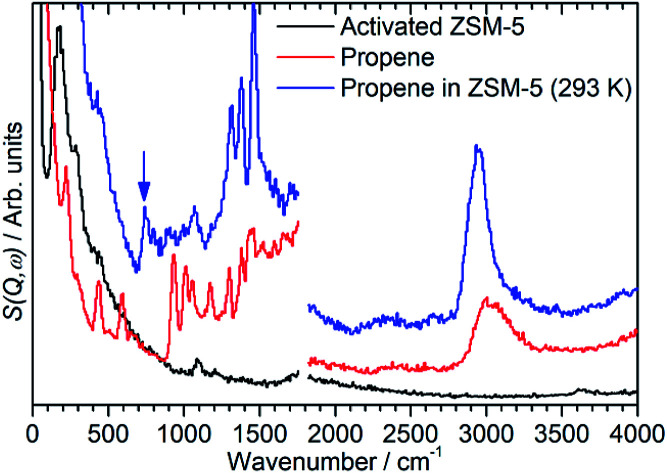

## 1. Introduction

Solid Brønsted acids such as zeolites are able to catalyse the oligomerization of light olefins such as propene to longer chain alkyl hydrocarbons.^1,2^ This reaction is of interest both as a means of C–C bond formation for the valorisation of light hydrocarbons and oxygenates and because it is the inverse reaction to the β-scission mechanism by which alkenes are cracked in commercial fluidised catalytic cracking (FCC) reactors and therefore has an effect on the final product composition of this important commercial process.^3,4^ The reaction proceeds *via* protonation of the olefin to a carbenium ion which reacts with other olefin molecules to give alkyl chains, with the exact composition being determined by shape selective effects from the zeolite pore.^5,6^ Oligomerization is particularly important at low temperatures: the small-pore zeolite ZSM-5 can convert propene even at room temperature, where it is reported to produce polypropylene chains of varying lengths,^5^ but above 473 K the dominant product is reported as mainly C_6_ isomers due to the increasing importance of β-scission reactions and isomerization to the equilibrium.^4^

This communication concentrates on the interaction of propene with ZSM-5 over the temperature range 140–373 K. In 1994 Spoto and co-workers used FTIR spectroscopy to investigate the oligomerization of ethene and propene on ZSM-5 at room temperature, noting that the confined structure of the zeolite influenced the degree of branching, distribution and length of the products.^5^ In the specific case of propene, branching was observed with approximately equal concentrations of CH_3_ and CH_2_ groups.^5^ In 1996, as part of a wider study, Chen and Bridger used ^13^C NMR spectroscopy to examine the ZSM-5/propene reaction system at 473 and discovered that when the oligomerization took place inside the ZSM-5 channels the products were nearly linear, with a small amount of methyl branching.^7^ A higher degree of methyl branching was observed at 503 K.^7^ In 2013 Corma and co-workers considered propene oligomerisation over ZSM-5 as part of the route to high quality liquid fuels and reported optimum diesel production to be correlated with Brønsted acid site concentration and the distribution of those sites within the crystal, as well as an adequate combination of micro- and mesoporosity; the latter parameters being influenced by crystal size.^8^ Supporting this perspective, Bernauer and co-workers highlight the importance of proton proximity in controlling adsorption, desorption and activity in propene oligomerization over ZSM-5; a control parameter accessible *via* manipulation of the zeolite's Si : Al ratio.^9^

In this paper, the ZSM-5 catalysed oligomerization of propene is studied by inelastic neutron scattering spectroscopy (INS) in order to obtain the vibrational spectrum of the reacted zeolite over an extended spectral range. INS provides clear visibility of low-energy hydrocarbon modes without interference from the zeolite framework and the use of a direct-geometry neutron instrument enables the collection of spectra with useful resolution in the 50–4000 cm^−1^ region by using multiple incident neutron energies.^10^ As expected, propene oligomerisation is observed at 293 K but, in contrast to the aforementioned FTIR study,^5^ only linear alkyl chains are observed. The difference in outcomes is attributed to differences in the size of the ZSM-5 crystallites, with this work establishing that confinement within the zeolite pore structure of the ZSM-5 crystallites constrains the oligomeric process, so that an end-to-end polymerisation stage is favoured. Minimal substrate/adsorbate interaction is observed for adsorption at cryogenic temperatures (140 K). Thus, the work confirms a role for zeolite crystallite size, a controllable parameter within the catalyst preparative stage, in being able to influence the product composition in technically relevant olefin oligomerization reactions.

## 2. Experimental

### 2.1 Catalyst

The ZSM-5 was sourced as a commercial powder-form zeolite catalyst supplied by Johnson Matthey (Si : Al ratio 30 : 1, BET surface area 370 m^2^ g^−1^). Scanning electron microscopy (Zeiss SEM300) revealed crystal sizes in the 0.2–1.0 μm range; typical crystallite size dimensions being 0.5 × 0.1 × 0.1 μm. The sample was calcined in air for 12 hours at 773 K to remove residual template material, followed by drying for three hours at 623 K under flowing helium (1000 sccm, CK gas, >99.999%) using a gas handling rig at the ISIS Facility, which is described elsewhere.^11^ The dried catalyst was transferred to an argon glovebox (MBraun UniLab MB-20-G, [H_2_O] < 1 ppm, [O_2_] < 1 ppm) and used to prepare samples for INS analysis as detailed below.

### 2.2 ZSM-5 + propene at 293 and 373 K

An 11.8 g sample of the pre-treated zeolite was loaded into an aluminium INS sample cell with an in-beam geometry of a 50 × 50 × 10 mm flat plate: this cell possesses gas handling fittings to allow introduction of gaseous adsorbates, indium wire gasket seals to allow use at cryogenic temperatures and top- and bottom-mounted electrical resistance heaters and thermocouples to allow accurate control of the sample temperature during measurement. Cooling to cryogenic temperatures was accomplished using the closed-cycle refrigerator that forms part of the MAPS sample environment. The loaded sample container was mounted on a gas handling centre stick and inserted in MAPS: a background spectrum of the unloaded zeolite was collected at <25 K with the instrument settings detailed above. The sample was removed and allowed to warm to room temperature to prevent condensation of the propene in the gas lines. A container of known volume was charged with 2.5 × 10^−2^ moles of propene, equating to a loading of 12.2 propene molecules per unit cell, and connected to the sample cell using a gas handling manifold. The propene was left connected to diffuse into the cell for 20 minutes: monitoring of the line pressure on the manifold showed that all propene had been adsorbed within 5 minutes. The loaded sample was returned to MAPS and spectra taken at the same conditions as for the background scans. After collection the sample was removed again and placed in a tube furnace which was used to heat the sample to 373 K for 1 hour to check for additional reactions at this temperature: no rise in the manifold line pressure was observed during heating, indicating that no propene remained in the gas phase prior to starting this process. The heated sample was allowed to cool before being returned to MAPS and the data collection process repeated.

### 2.3 Propene reference spectrum

A model spectrum of solid propene was obtained for comparison purposes by introducing 4.076 × 10^−2^ moles of propene into a sample cell of known volume designed for the INS measurement of gaseous compounds. The bottom portion of this can was immersed in liquid nitrogen to condense all of the propene into the portion of the cell which lies within the neutron beam when installed. Following condensation, the propene sample was installed on MAPS and the spectra collected.

### 2.4 ZSM-5 + propene: low temperature studies

To obtain INS spectra of the cryogenically-loaded propene in ZSM-5 a second zeolite sample of mass 11.4 g was loaded into another aluminium sample can of the same design used previously. After collection of a background spectrum, as before, this cell was allowed to warm to room temperature to prevent propene condensation in the gas lines and then immersed in liquid nitrogen up to the level of the top of the catalyst bed. 1.3 × 10^−2^ moles/6.6 molecules per unit cell of propene were introduced using the gas manifold and allowed to freeze into the cooled portion of the cell. The sample was returned to MAPS and slowly heated to 140 K using the cell-mounted heaters to allow the propene to melt and diffuse into the zeolite as a liquid. Following 1 hour at 140 K for diffusion the sample was returned to <25 K and the spectra of the cryogenically loaded propene collected.

### 2.5 Inelastic neutron scattering

**Fig. 1:**
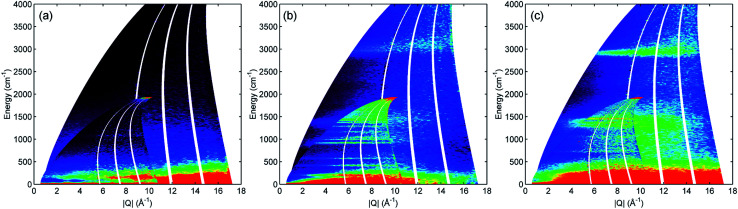
*S*(*Q*,*ω*) mitre plots recorded on MAPS at incident energies of 5244 cm^−1^ and 2017 cm^−1^ (insets). Heat map represents scattered neuron intensity in arbitrary units. (a) Activated ZSM-5 reference; (b) pure propene reference; (c) propene adsorbed into ZSM-5 at 293 K.

**Fig. 2:**
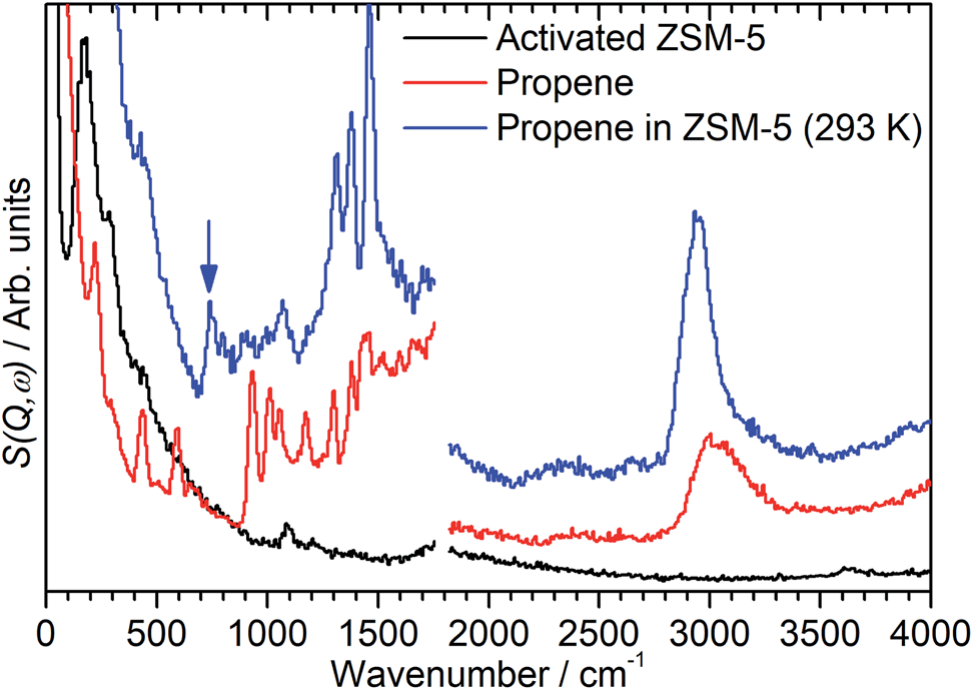
INS spectra of ZSM-5 before (black) and after (blue) addition of propene at 293 K compared to reference samples of pure propene (red). Spectra recorded on the MAPS spectrometer at incident energies of 2017 (left) and 5244 (right) cm^−1^ and integrated over the momentum transfer range 0 ≤ *Q* ≤ 10 Å^−1^. The pure propene reference spectrum (red) is scaled to correct for the different number of moles of propene in the sample. The blue arrow indicates the presence of an in-phase methylene rock in the chemisorbed spectrum at 736 cm^−1^.

**Fig. 3:**
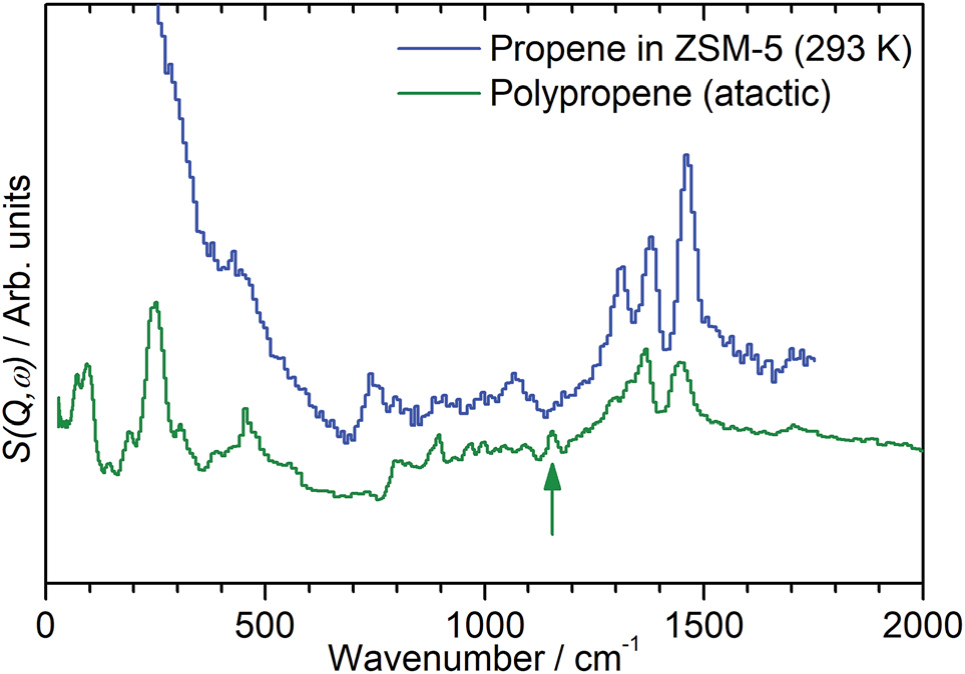
Comparison of INS spectra of propene in ZSM-5 (blue) against a reference spectrum for atactic polypropene (green). The branching –CH_3_ wag mode of the polypropylene is highlighted (green arrow). Propene/ZSM-5 spectrum recorded on the MAPS spectrometer at an incident energy of 2017 cm^−1^ and integrated over the momentum transfer range 0 ≤ *Q* ≤ 10 Å^−1^. Polypropene spectrum sourced from the ISIS INS database and recorded on the indirect geometry spectrometer TOSCA.^21,24^ Comparison made at low energies only due to TOSCA exhibiting poor resolution above ∼2000 cm^−1^. Spectra offset in *y*-axis for clarity.

**Fig. 4:**
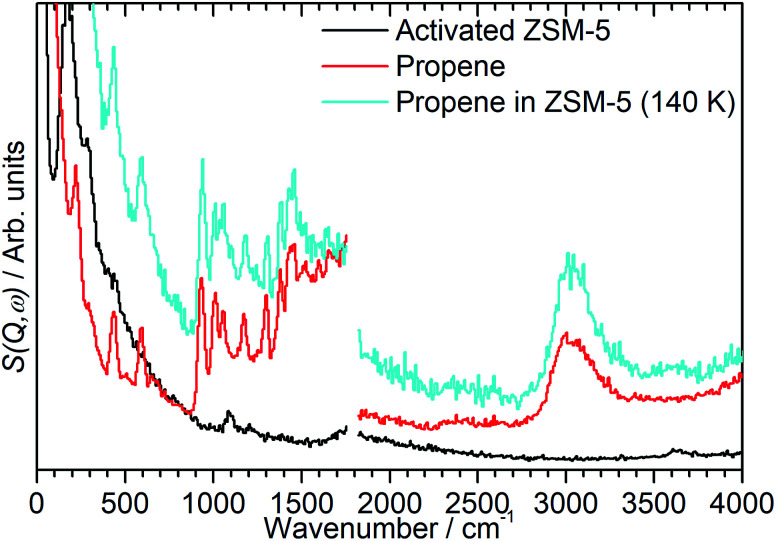
INS spectra of ZSM-5 before (black) and after addition of propene at 140 K (turquoise) compared to a reference sample of pure propene (red). Spectra recorded on the MAPS spectrometer at incident energies of 2017 (left) and 5244 (right) cm^−1^ and integrated over the momentum transfer range 0 ≤ *Q* ≤ 10 Å^−1^. The pure propene reference spectrum (red) is scaled to correct for the different number of moles of propene in the sample.

**Fig. 5:**
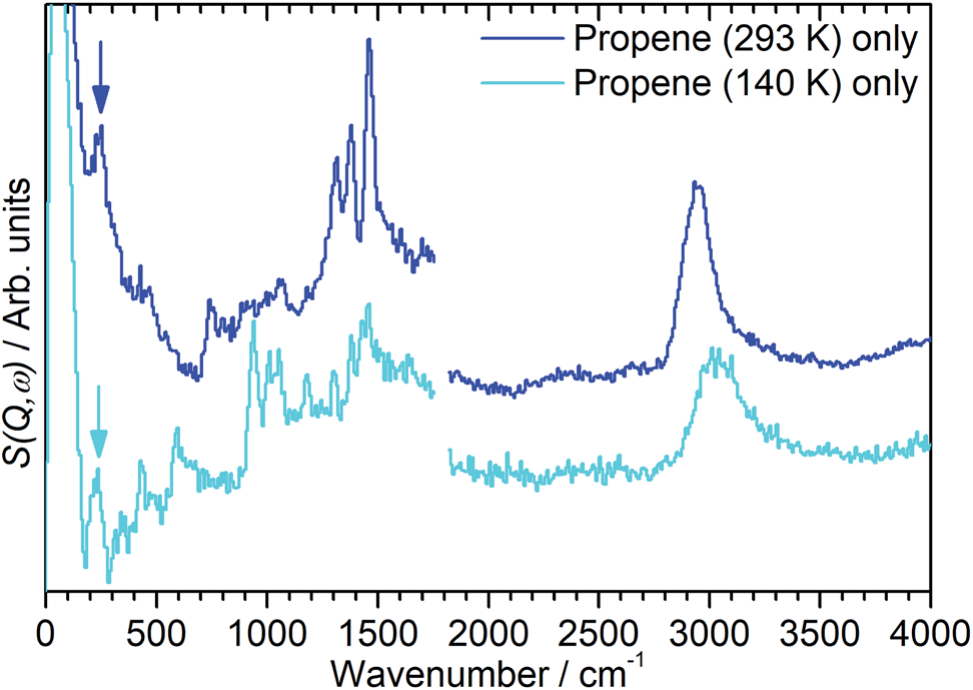
INS spectra of propene adsorbed into H-ZSM-5 at temperatures of 293 K (blue) and 140 K (turquoise) following subtraction of the INS spectra of the zeolite background, showing the presence of methyl torsion peaks at ∼230 cm^−1^ (highlighted) which are masked by the zeolite contributions in Fig. 2 and 3. Spectra recorded on MAPS at incident energies of 2017 (left) and 5244 (right) cm^−1^ and integrated over the momentum transfer range 0 ≤ *Q* ≤ 10 Å^−1^. The spectra are scaled to correct for differences in the number of moles of propene in each sample and offset in the *y*-axis for clarity.

All INS spectra reported here were collected on the direct geometry spectrometer Multi-Angle Position Sensitive (MAPS) at the ISIS neutron source facility using the high resolution A-chopper package at incident energies of 5244 and 2017 cm^−1^, with chopper frequencies of 600 and 400 Hz respectively. Time-of-flight data reduction and analysis of all collected datasets was performed using the Mantid software package.^12^ Generation of the mitre plots presented in Fig. 1 and the integrated plots in Fig. 2–5 made use of Mslice.^13^ The intensities of the spectra in the integrated plots were scaled using Mantid to correct for the different number of moles of propene in each sample.

## 3. Results

### 3.1 ZSM-5 + propene at 293 and 373 K

INS is an inherently 2-dimensional spectroscopic technique, offering information on neutron scattering intensity (*S*) with respect to both energy transfer (*ω*, cm^−1^) and momentum transfer (*Q*, Å^−1^). On direct geometry instruments, where the full range across both dimensions is accessible, the conventional way to present the full *S*(*Q*,*ω*) spectrum is as a 2-dimensional ‘mitre’ plot.^14^Fig. 1 shows the mitre plots for propene adsorbed into ZSM-5 at 293 K (Fig. 1c) compared with those for reference samples of activated ZSM-5 (Fig. 1a) and propene (Fig. 1b). Due to the low scattering cross-sections of Si and Al and the low number of acid sites in the zeolite the modes in the activated ZSM-5 plot are extremely weak, while the greater intensity of the hydrocarbon samples due to the increased number of ^1^H scattering centres is readily apparent. The propene spectrum (Fig. 1b) shows a clear signal in the C–H stretch region around 3000 cm^−1^ and two groups of bands in the deformation region of the spectrum below 1600 cm^−1^. The spectrum of the combined sample (Fig. 1c) shows changes consistent with oligomerization, namely the shifting of the C–H stretch signal into a more intense band over a narrower energy distribution due to elimination of sp^2^ carbon centres and the replacement of the narrow, intense signals in the deformation region with a larger number of weaker signals due to the replacement of a few well-defined C=C motions by multiple methylene modes.

By integrating the *S*(*Q*,*ω*) plot with respect to *Q* it is possible to reduce it to the more familiar 2D spectrum similar to that generated by optical spectroscopic methods. Limiting the *Q* range so that the integration is performed over to the lower values of *Q* ≤ 10 Å^−1^ also has the useful effect of eliminating a large proportion of the contributions to the scattering from overtone modes and phonon wings which predominantly scatter at large *Q* values, as can be seen in Fig. 1.^10,14^ The integrated spectra for the mitre plots in Fig. 1 are presented in Fig. 2. The modes in the zeolite spectrum are still weak but the concentration of the signal over the integrated range allows the identification of an O–H stretch mode at 3590 cm^−1^ and O–H deformation features at 1080 and 100–350 cm^−1^.^15^ The propene spectrum exhibits multiple, clearly defined peaks which were assigned based on values reported in the literature.^16–19^ The most important peaks of note are the methyl torsion at 220 cm^−1^, the C=C–C scissors at 429 cm^−1^, the =CH_2_ twist and rock at 585 and 915 cm^−1^ and the shape of the C–H stretch as a broad peak centred at 3000 cm^−1^ due to equal contributions from sp^2^ and sp^3^ C–H bonds. The shifting of some of these bands relative to their literature values is due to this spectrum being collected at low temperature and in the solid phase.

The spectrum of the combined sample (Fig. 2, blue) confirms that the changes observed in the mitre plot correspond to the catalytic oligomerization of the propene by the zeolite acid sites. The C=C modes highlighted above are completely suppressed in the combined spectrum and are replaced by a broad band of overlapping methylene modes in the 700–1100 cm^−1^ range. The three peaks at 1312, 1376 and 1464 cm^−1^ are assigned to a new methylene scissors peak and the symmetric and asymmetric deformation of the terminal methyl groups respectively. The shift in location and narrowing of the C–H stretch peak to a new centre at 2945 cm^−1^ indicates the removal of essentially all sp^2^ carbon centres by the chain formation reaction: the residual intensity above 3000 cm^−1^ in the combined spectrum is due to a combination mode between the sp^3^*ν*(C–H) vibrations and the transverse acoustic modes of the oligomer chain, which we have previously observed in other linear saturated oligomers and in polyethylene.^20^ Finally, the combined spectrum (Fig. 2, blue) contains no sign of the zeolite O–H stretch indicating the consumption of a majority of the acid sites to initiate the oligomerization reaction. The torsion peak of the terminal methyl groups is still present in the combined spectrum but is masked by the contributions from the zeolite framework deformations: subtraction of the zeolite contribution to give the propene contribution alone (presented as Fig. 5, below) clearly shows this mode at 226 cm^−1^. To confirm that this spectrum represents a fully oligomerized sample, additional spectra were collected after heating the sample to 373 K: no changes were observed, indicating that the reaction runs to completion at 293 K.

**Scheme 1:**
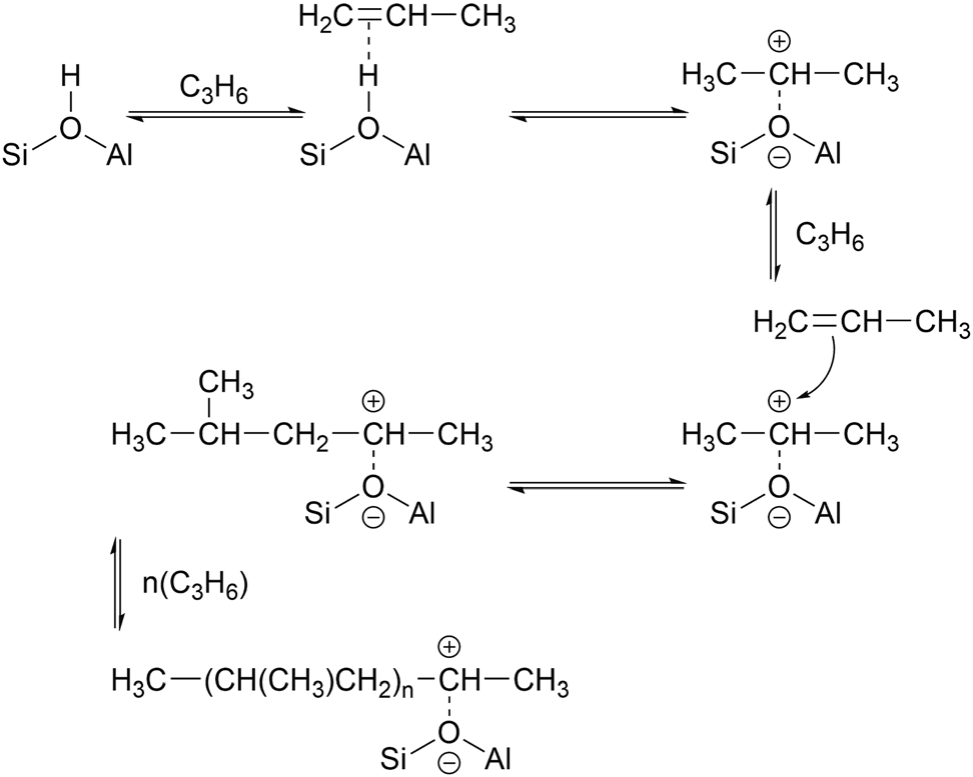
Formation of branched polypropene oligomers at acid sites without confinement.

**Scheme 2:**
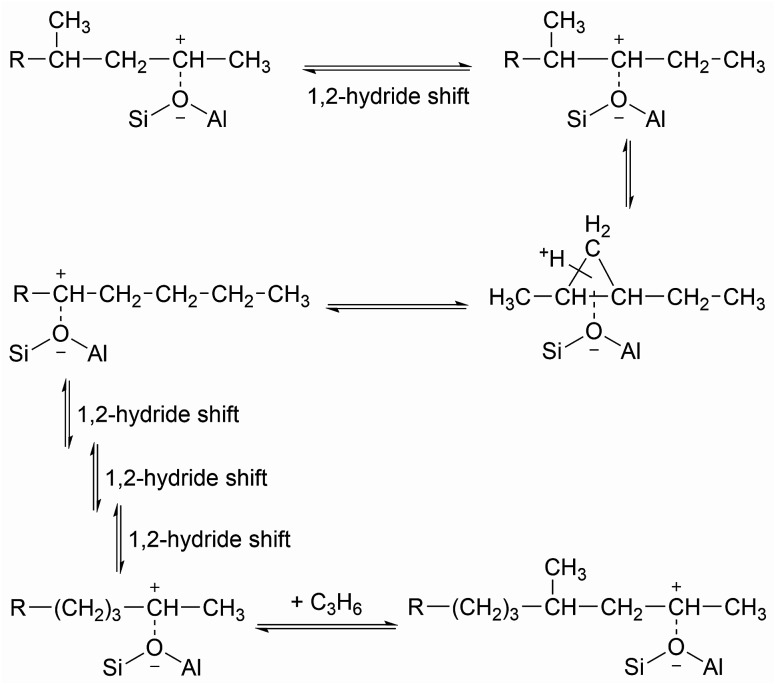
Rearrangement to form linear oligomer and subsequent chain propagation favoured for acid sites within the zeolite pore structure, adapted from ref. 20.

While the evidence of oligomerization is clear, the combined spectrum contains no evidence of any branching in the oligomerized product. No peaks attributable to –CH– groups are visible and the –CH_3_ wag mode at 1150 cm^−1^ is also absent (Fig. 3). The large number of overlapping methylene modes is also suggestive of long alkyl chains and not found in the spectra of the various polypropylene tacticities.^14,21^ In particular, the strong signal at 736 cm^−1^, highlighted in Fig. 2, is characteristic of the in-phase methylene rock in chains of adjacent CH_2_ groups of length ≥ C_4_.^22^ As a result, we conclude that the confinement in the ZSM-5 pores results in the oligomerization reaction proceeding *via* an end-to-end mechanism to produce linear alkyl species. This conclusion differs from that obtained from infrared studies by Spoto *et al.*,^5^ who deduced from analysis of the *ν*(C–H) region (3000–2800 cm^−1^) that the oligomeric chains formed from propene in H-ZSM-5 at room temperature have a branched structure with approximately equal concentrations of CH_3_ and CH_2_ groups, generated by the mechanism shown in Scheme 1. We note however that the ZSM-5 catalyst used by Spoto *et al.* comprised unusually small zeolite crystals (20–50 nm) with a correspondingly very high external surface area, as confirmed by the high relative intensity of the silanol *ν*(O–H) infrared band at 3740 cm^−1^ due to external silanol groups. We suggest therefore that the room temperature oligomerization is more sterically constrained within the internal pores in our catalyst (average crystal size 0.5 × 0.1 × 0.1 μm). We emphasise here in particular the detection by INS of the in-phase methylene rock at 736 cm^−1^ as evidence for linear chains which cannot be seen in the infrared experiments. This primarily linear product does match with the results derived from solid state ^13^C NMR studies by Chen and Bridger^7^ for the oligomerization of alkenes in ZSM-5 crystals which have had the surface acid groups selectively deactivated. They propose a mechanism whereby the branched carbenium product of propene addition undergoes rearrangement to incorporate the methyl side branch within the chain backbone prior to subsequent addition of further propene molecules. This rearrangement proceeds through a protonated cyclopropyl intermediate, as shown in Scheme 2, and is driven by steric effects from the confinement within the zeolite pore favouring chains with a smaller diameter and the higher heat of adsorption for linear alkyl chains within ZSM-5.^23^ It is believed that this work reports the first evidence for this reaction mechanism obtained through vibrational spectroscopy.

### 3.2 ZSM-5 + propene: low temperature studies

In order to investigate the possible effect of interactions between the adsorbed hydrocarbon and the zeolite pore wall on the INS spectrum a zeolite sample was loaded with propene at cryogenic temperatures to ensure no reaction occurred. A sample of activated zeolite was cooled to 77 K by immersion in liquid nitrogen and the propene introduced from the top of the sample cell. The sample was then warmed to 140 K to allow the propene to diffuse through the zeolite as a liquid, followed by cooling to <30 K for INS measurement. The resulting spectrum (Fig. 4) retains all the features of the propene reference spectrum with the exception of the methyl torsion peak at *ca.* 216 cm^−1^, which is obscured by the zeolite contribution unless this is subtracted as for the oligomerized sample (Fig. 5), and it is therefore clear that the propene is stable within the zeolite under these conditions. The zeolite O–H stretch is also still perceptible in the combined spectrum (Fig. 4, turquoise), again indicating that no protonation reactions have occurred, although the centre of the peak appears to have shifted to a slightly lower energy: if this is the case it indicates that the hydrogen-bonded olefins which are reported as a precursor to the protonation reaction can still form at this temperature.^5^ Studies are underway to further characterise the nature of the oligomerisation process over the temperature range 140–293 K.

## 4. Conclusions

INS has been used to examine the reaction between propene and an activated sample of ZSM-5 at 140, 293 and 373 K; the following conclusions can be drawn.

• At 293 K propene oligomerises within the zeolite, proceeding *via* an end-to-end mechanism (Scheme 2) to produce linear alkyl species.

• The formation of linear alkyl species in preference to formation of branched structures is attributed to confinement within the zeolite pore structure of the particular ZSM-5 crystallites examined in this work.

• The reaction of ZSM-5 and propene at 373 K yielded the same spectrum as that observed for reaction at 293 K, indicating that the oligomerisation process is complete at 293 K.

• The INS spectrum of propene exposed to ZSM-5 at 140 K reproduces the reference spectrum of molecular propene, indicating minimal adsorbate/substrate interaction at this temperature.

## Conflicts of interest

There are no conflicts to declare.

## Supplementary Material
